# The effects of immune protein CD3ζ development and degeneration of retinal neurons after optic nerve injury

**DOI:** 10.1371/journal.pone.0175522

**Published:** 2017-04-25

**Authors:** Tao He, Xavier Mortensen, Ping Wang, Ning Tian

**Affiliations:** 1 Eye Center Remin Hospital of Wuhan University Wuhan, Hubei, PR China; 2 Department of Ophthalmology and Visual Science John Moran Eye Center University of Utah School of Medicine, Salt Lake City, UT, United States of America; 3 VA Salt Lake City Health Care System, Salt Lake City, UT, United States of America; Instituto Murciano de Investigacion y Desarrollo Agrario y Alimentario, SPAIN

## Abstract

Major histocompatibility complex (MHC) class I molecules and their receptors play fundamental roles in neuronal death during diseases. T-cell receptors (TCR) function as MHCI receptor on T-cells and both MHCI and a key component of TCR, CD3ζ, are expressed by mouse retinal ganglion cells (RGCs) and displaced amacrine cells. Mutation of these molecules compromises the development of RGCs. We investigated whether CD3ζ regulates the development and degeneration of amacrine cells after RGC death. Surprisingly, mutation of CD3ζ not only impairs the proper development of amacrine cells expressing CD3ζ but also those not expressing CD3ζ. In contrast to effects of MHCI and its receptor, PirB, on other neurons, mutation of CD3ζ has no effect on RGC death and starburst amacrine cells degeneration after optic nerve crush. Thus, unlike MHCI and PirB, CD3ζ regulates the development of RGCs and amacrine cells but not their degeneration after optic nerve crush.

## Introduction

Retinal ganglion cells (RGCs) are vulnerable in eye diseases and different subtypes of amacrine cells respond to RGC injury differently. For instance, injury of RGC axons in neonatal rats or RGC elimination in ferret altered the number of GABAergic and glycinergic amacrine cells [1–3] without changing the number of dopaminergic amacrine cells, cholinergic amacrine cells (or starburst amacrine cells, SACs), and substance P-positive amacrine cells [4–6]. In addition, monkeys, mice and rats with experimental glaucoma lose amacrine cells expressing glycine, tyrosine hydroxylase, GABA, vesicular acetylcholine transporter (VAChT), choline acetyltransferase (SACs), NADPH-diaphorase and nitric oxide synthase [3, 7–9]. Furthermore, rats have substantial reduction in amacrine cells expressing arvalbumin, glycine transporter, and choline acetyltransferase (SACs) with retinal ischemia [10]. However, all previous studies focused on amacrine cell death or changes of gene expression after RGC injury. To our knowledge, there is no study on the dendritic reorganization of amacrine cells after RGC death. Because proper dendritic structure of amacrine cells is critical to the maintenance of a functional synaptic circuitry in the retina, one goal of this study is to determine whether RGC death leads to changes of the dendritic structure and density of starburst amacrine cells although these cells seem to be resistant to RGC death caused by RGC axonal injury.

Recent studies have shown that immune molecules are expressed by neurons and play fundamental roles in the development and pathogenesis of the nervous system. Mice with defected MHCI or its putative receptors, PirB or T-cell receptor (TCR), have abnormal retinogeniculate connections, abnormal motor learning and abnormal synaptic plasticity in the visual cortex [11–13]. In addition, MHCI-deficient mice have reduced regeneration of axons and more extensive loss of synapses on motor neurons after injury [14–16] while up-regulated MHCI expression in neurons significantly promoted the recovery of locomotor abilities after spinal cord injury in mice [17]. However, a recent report showed that mice with MHCI and PirB knockout have smaller infarcts and enhanced motor recovery in a stroke model, less cell death after ischemia of the hippocampus, and reduced reactive astrocytic response after middle cerebral artery occlusion [18]. Thus, the roles of MHCI and its receptors on pathogenesis and protection of neurons appear contradictory.

In the immune system, T-cell receptor (TCR) functions as MHCI receptor [19]. In the retina, both MHCI and the key component of TCR, CD3ζ, are expressed by retinal neurons, including RGCs and displaced starburst amacrine cells (DSAC) [11, 20]. Genetic mutation of these molecules compromised the development of RGC dendrites and axonal projections [11, 20]. Another goal of this study is to determine whether mutation of CD3ζ affects the development of SACs/DSACs and regulates the degeneration of these cells after optic nerve crush (ONC). Accordingly, we qualitatively examined the number and the dendritic structure of SACs/DSACs of mice with or without mutation of CD3ζ before and after ONC.

## Materials and methods

### Animals

Thy1-Stop-YFP (yellow fluorescent protein), B6;129-Chat^tm1(cre/ERT)Nat^/J (ChAT-CreER) and B6.129S4-Cd247^tm1Lov^/J (CD3ζ-/-) mice are all on C57BL/6 background. Thy1-Stop-YFP mice were obtained from Dr. Joshua Sanes’ laboratory at Harvard University [21]. ChAT-CreER [22] and CD3ζ-/- [23] were obtained from The Jackson Laboratory (Bar Harbor, ME). The ChAT-CreER mice were bred into the Thy1-Stop-YFP mice to generate ChAT-CreER:Thy1-Stop-YFP double transgenic mice and YFP is expressed specifically in SACs/DSACs upon IP injection of Tamoxifen. These mice served as wild type controls (WT). ChAT-CreER:Thy1-Stop-YFP:CD3ζ-/- triple transgenic mice were generated by breeding ChAT-CreER:Thy1-Stop-YFP mice with CD3ζ-/- mice. In these triple transgenic mice, the gene (CD247) encoding the protein CD3ζ is mutated. All mice were treated with IP injection of Tamoxifen (200 μg) around P30. All animal procedures and care were preformed following protocols approved by the IACUC of the University of Utah and the IACUC of VA Salt Lake City Health Care System in compliance with PHS guidelines and with those prescribed by the Association for Research in Vision and Ophthalmology (ARVO).

### Optic nerve crush procedure

The ONC procedure was performed unilaterally on all mice around the age of P90. The animals were deeply anesthetized with 2–5% isoflurane (MWI, Meridian, ID) using a computerized mouse anesthesia suite (SomnoSuite® System, Kent Scientific Corporation, Torrington, CT) and a local application of 0.5% proparacaine hydrochloride ophthalmic solution (Falcon Pharmaceuticals, Fort Worth, TX). Under a stereo surgical microscope, a small cut was made at the lateral canthus of eyelid to expose the lateral side of eyeball. Then, a small incision was made in the conjunctiva beginning inferior to the eyeball and around the cornea temporally. With micro-forceps, held the edge of the conjunctiva next to the eyeball and retracted it. Gently deflected the orbital muscles and rotated the eyeball nasally to exposes the posterior aspect of the eyeball and optic nerve. Using a Dumont #N7 cross-action forceps (cat. #RS-5027; Roboz) to hold the optic nerve at about 1 mm from the back of eyeball for 10 seconds, with only pressure from the self-clamping action of the forceps to press on the nerve. The Dumont cross-action forceps has its own spring action, which applies a constant and consistent force to the optic nerve. After 10 seconds the optic nerve was released and the forceps were removed to allow the eyeball to rotate back into place. A small amount of surgical lubricant (KY jelly; McNeil-PPC, Skillman, NJ) was applied to the eye to protect it from drying and a subcutaneous injection of buprenorphine was administered for post-operative pain control. The mouse was placed on a warming pad and monitored until it fully recovered from anesthesia. For the first three days after the procedure, systemic analgesics (buprenorphine) and topical antibiotic ointment were applied twice daily and the mouse was closely monitored for possible infection, bleeding, and loss of muscular control [24–26]. The effectiveness of the injury of RGC axons was confirmed by CTB labeling of the optic nerve 1 week after the ONC.

### Primary antibodies

Rabbit polyclonal antibody against green fluorescent protein (GFP) conjugated with AlexaFluor 488 was purchased from Molecular Probes (Eugene, OR; catalog No. A21311). This antibody was raised against GFP isolated directly from *Aequorea Victoria* and has been characterized by immunocytochemistry in granule cells [27], olfactory sensory neurons [28], and hipocampal neurons that express GFP [29]. Antibody directed toward choline acetyltransferase (ChAT) was purchased from Millipore (Temecula, CA; catalog No. AB144P). This polyclonal antibody was raised in goat against human placental enzyme and has been characterized by Western blotting, recognizing a band at 68–70 kD. Mouse monoclonal antibody to rat CD3ζ Clone IF4 (CALTAG Laboratories) was purchased from Invitrogen (Invitrogen Corporation, Camarillo, CA; catalog No. MR5300). This antibody has been characterized by immunocytochemistry of rat T-cells [30]. The fluorescent dye, DAPI (4',6-Diamidino-2-Phenylindole, Dihydrochloride), for nuclear stain was purchased from Molecular Probes-ThermoFisher Scientific (Catalog No. D1306). The secondary antibody was purchased from Jackson Immune Research Laboratories (West Grove, PA).

### Preparation of retinal whole-mounts and retina sections for fluorescent imaging

SACs/DSACs were imaged on whole mount retinal preparation while the dendritic ramification of these cells in the inner plexiform layer (IPL) was imaged on retinal slice preparation using confocal microscopy. The procedures for fluorescent immuno-labeling of YFP-expressing retinal neurons on retinal whole-mounts and slide preparations have been described previously in detail [20, 31–32]. In brief, mice were euthanized with 100% CO_2_ followed by cervical dislocation. For retinal whole mount preparation, retinas were isolated and fixed in 4% paraformaldehyde (PFA) in 0.01M phosphate-buffered saline (PBS; pH 7.4) for 30 minutes at room temperature. Fixed retinas were washed 10 min × 3 in 0.01 M PBS and incubated in blocking solution (10% normal donkey serum) at 4°C for 2 hours. Next, retinas were incubated in rabbit polyclonal anti-GFP antibody conjugated with Alexa Fluor488 (1:500) and goat polyclonal anti-ChAT antibody (1:150) for 7 days at 4°C. Following this retinas were incubated in an Alexa 647-conjugated donkey anti-goat (1:100) secondary antibody overnight at 4°C, washed 3 x 10 min, and then placed in DAPI (0.3–3 µM) solution overnight at 4°C. Retinas were then washed and flat mounted on Super-Frost slides (Fisher Scientific, Pittsburgh, PA) with Vectashield mounting medium for fluorescence (Vector Laboratories, Burlingame, CA).

For retina section preparation, the whole eyes were removed and fixed in 4% paraformaldehyde (PFA) for 2 hours. Fixed eyes were washed 3 times for 10 minutes each in 0.01 M PBS, moved to a 15% sucrose solution for 1.5 hours at room temperature, and then incubated in 30% sucrose at 4°C overnight. Eyes were than embedded in Tissue-Tek OCT compound (Sakura Finetek USA, Torrance, CA), and stored at -80°C until ready for sectioning. Whole eyes were sectioned vertically with a thickness of 12–15 µm using a Leica CM-3050S cryostat microtome (Leica Biosystems, Wetzlar, Germany), and collected on Super-Frost Plus slides (Fisher Scientific, Pittsburgh, PA). A rabbit polyclonal anti-GFP antibody conjugated with Alexa Fluor488 (1:500) was used to label YFP-expressing starburst amacrine cells and a goat polyclonal anti-ChAT antibody (1:150) was used to label all starburst amacrine cells, respectively. An Alexa 647-conjugated donkey anti-goat (1:100) secondary antibody was used to reveal the anti-ChAT bindings.

### Confocal laser scanning microscopy

Fluorescent images were collected with a dual-channel Zeiss microscope (Carl Zeiss AG, Germany) with the C-Apochromat 40x 1.2 W Korr water immersion lens. Image stacks of YFP-expressing SACs/DSACs in whole-mount retinas were collected at intervals of 0.5 µm. IPLab software (Scanalytics, Inc., Fairfax, VA) was used to align multistacks of images together. Quantitative dendritic analysis of YFP-expressing SACs/DSACs was carried out by using Neurolucida software (Neurolucida 2000, Microbrightfield, Williston, VT). Dendritic trees of SACs/DSACs were reconstructed by using Neurolucida software [20, 33]. The intensity and contrast of some images were adjusted using the software ImageJ (NIH).

### Statistical analysis

Data are all presented as mean ± SEM in the text, figures and tables (Igor Pro, WaveMetrics, Inc., Lake Oswego, OR). Student t-tests were used to examine the difference between two means using Statview (Abacus Concepts, Berkeley, CA, USA).

## Results

### The dendritic structure of SACs/DSACs was qualified

**Fig 1:**
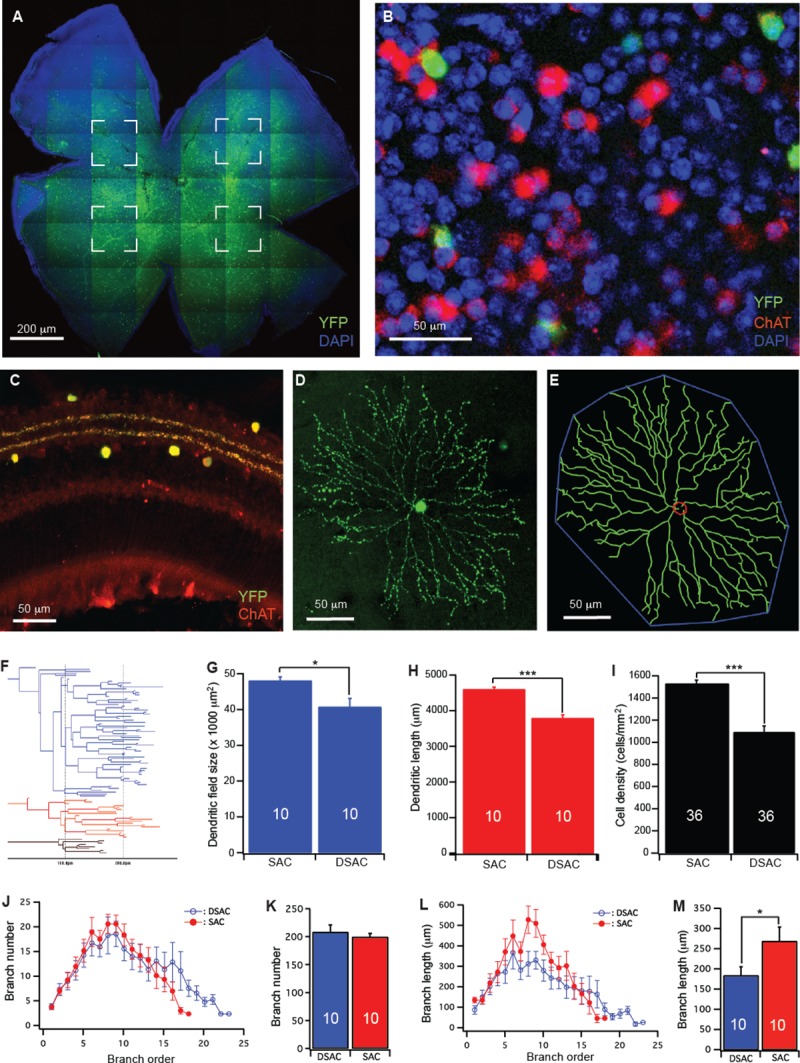
Quantify dendritic structure of mouse SACs/DSACs. (**A**) Whole mount retina of a CreER-ChAT:Stop-YFP mouse stained with anti-GFP (green) and DAPI (blue). The four dashed-line boxes indicate the areas used for cell density calculation. (**B**) An enlarged area of the retinal ganglion cell layer showing YFP staining of Cre activated DSACs (green), anti-ChAT antibody staining of all DSACs (red) and DAPI (blue) staining of the GCL. (**C**) A cross section of the retina of a CreER-ChAT:Stop-YFP mouse showing YFP staining of Cre activated SACs and DSACs (green) and anti-ChAT antibody staining of SACs and DSACs (red) in the retina. (**D**) A maximum projection of the dendrites of a DSAC. (**E**) The tracing results of the DSAC shown in panel D (green, dendrites; red, soma; blue, dendritic field). (**F**) The dendrogram of the DSAC shown in panel D. The total length of dendrites, the number of dendritic branches, the order of each dendritic branch and the length of each dendritic branch were derived from this dendrogram. (**G**) Average size of dendritic field of SACs and DSACs. (**H**) Average length of dendrites of SACs and DSACs. (**I**) Average density of SACs and DSACs. (**J**) The number of dendritic branch as a function of dendritic order of SACs and DSACs. (**K**) The average number of dendritic branch of SACs and DSACs. (**L**) The length of dendritic branch as a function of dendritic order of SACs and DSACs. (**M**) The average length of dendritic branch of SACs and DSACs. The numbers in the columns of panels G, H, K and M indicate number of cells analyzed. The numbers in the columns of panel I indicate the numbers of images analyzed. In this figure and all following figures, * indicates 0.01<p<0.05; ** indicates 0.001<p<0.01; *** indicates p<0.001.

SACs/DSACs are the most abundant amacrine cells in mammalian retina. Their somas are located in both the GCL as displaced starburst amacrine cells (DSACs) [34–35] and the INL as conventionally placed starburst amacrine cells (SACs) [34, 36–37], respectively. Previous studies have demonstrated that optic nerve section of one eye of rats had no qualitative difference on SACs/DSACs [4] or any other cell type than RGCs in the GCL [6]. In order to label the dendrites of individual SACs/DSACs, we generated the CreER-ChAT:Stop-YFP double transgenic mice by breeding the ChAT-CreER mice into the Thy1-Stop-YFP mice. By double labeling of ChAT and YFP, we show that all YFP-expressing cells (green) in the retina of CreER-ChAT:Stop-YFP mice are ChAT positive (red), demonstrating that YFP is expressed by both SACs and DSACs in these mice (Fig 1A–1C).

We then quantified the dendritic structure of SACs and DSACs on confocal image stacks through dendritic tracing using Neurolucida (Neurolucida 2000, Microbrightfield, Williston, VT) [20, 31, 33]. Fig 1D shows a maximum projection of the dendrites of a DSAC and Fig 1E shows the tracing result of the cell shown in Fig 1D. The dendritic field (DF) size of each SAC/DSAC was measured by linking tips of dendritic arbors and then calculating the area inside (Fig 1E). The total length of dendrites, the number of dendritic branches, the order of each dendritic branch and the length of each dendritic branch were determined from the dendrogram (Fig 1F). To calculate the overall cell density in GCL and the density of SACs and DSACs, four image stacks with the size of 160 µm X 160 µm were taken from the four quarters of the retina approximately 200 µm away from the optic nerve head (Fig 1A). The overall cell density of GCL was calculated based on the number of DAPI stained cells in GCL while the densities of SACs and DSACs were determined based on the anti-ChAT stained cells in INL and GCL, respectively (Fig 1B). Our results showed that the DSACs have significantly smaller dendritic field size, shorter dendritic length and lower cell density than that of SACs (Fig 1G–1I). Further analysis of the dendritic structure of SACs and DSACs showed that, although DSACs have smaller dendritic field and shorter total dendritic length than that of SACs, they have more complex dendritic architecture as reflected by 14.5% more dendritic branches (Fig 1K) preferentially distal dendritic branches (Fig 1J), and 40% shorter average branch length (Fig 1M) preferentially proximal dendritic branches (Fig 1L. Also see S1 Table for more details of statistic tests).

### RGC death induced by ONC is associated with quick dendritic reorganization of SACs/DSACs

**Fig 2:**
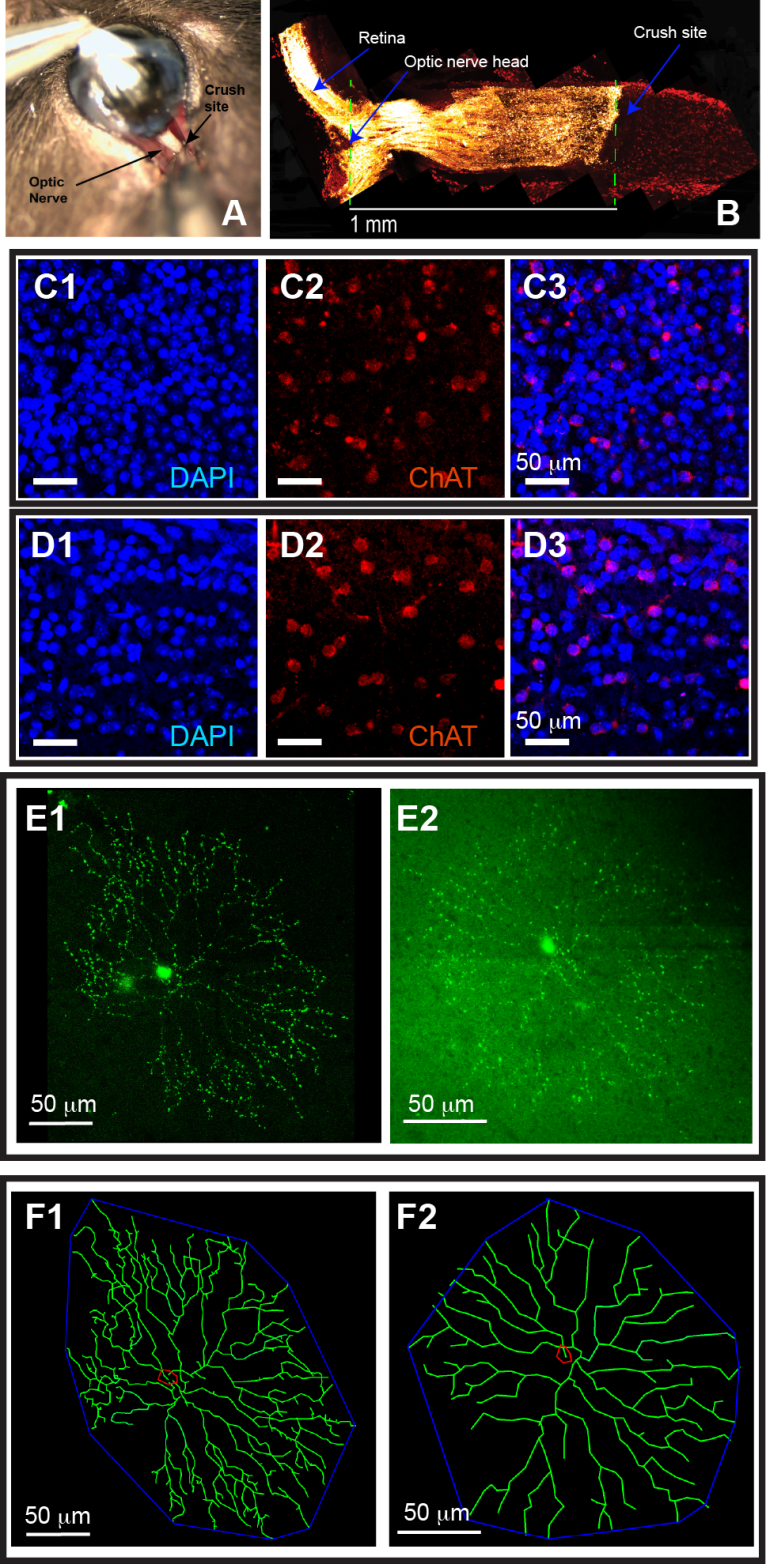
RGC death after ONC is associated with quick dendritic reorganization of SACs/DSACs. (**A**) Optic nerve exposing and crushing. (**B**) An image of the longitude cross section of the proximal portion of the optic nerve and the posterior portion of the eye. RGCs and their axons are labeled with CTB conjugated with Alexa Fluor 594 (yellow) and the section was co-labeled with DAPI (red). Please note that the axonal transport of CTB along RGC axons is completely blocked at the crush site. (**C**) Magnification from whole mount retina of a mouse without ONC showing the density of DAPI stained nuclei in the GCL (C1, blue), the density of anti-ChAT stained DSACs (C2, red) and the overlay of the DAPI and anti-ChAT stainings (C3). (**D**) Magnification from whole mount retina of a mouse with ONC showing the density of DAPI stained nuclei in the GCL (D1, blue), the density of anti-ChAT stained DSACs (D2, red) and the overlay of the DAPI and anti-ChAT stainings (D3). (**E**) Representative maximum projection images of DSACs without (E1) and with (E2) ONC. (**F**) The tracing results of the DSACs shown in panel E.

Although direct RGC injury by ONC or glaucoma seems not to cause SACs/DSACs death [1, 3–9, 38–40], it is unknown whether SACs/DSACs lose structural and functional integrity after RGC death. Accordingly, we specifically injured the axons of RGCs through ONC of CreER-ChAT:Stop-YFP mice. The ONC procedure was performed unilaterally on all mice around the age of P90. The crush was made about 1 mm from the back of eyeball (Fig 2A) and the effectiveness of the injury of RGC axons was confirmed by CTB (cholera toxin subunit B conjugated with Alexa Fluor 594, Invitrogen, Camarillo, CA, catalog No. C22842) labeling of the optic nerve 1 week after the ONC (Fig 2B). We then qualified the cell death in the GCL and the densities of SACs and DSACs after ONC to determine whether SACs/DSACs degenerate concurrently with the death of RGCs. Fig 2C and 2D show anti-ChAT antibody and DAPI stained nuclei and cells in the GCL of retinas of mice without (2C) and with (2D) ONC, respectively. It is evident that the number of DAPI stained nuclei in the GCL is significantly reduced 10 days after ONC while the number of DSACs of the same retina is not affected. In addition, we qualified the dendritic structure of SACs/DSACs 7 and 10 days after ONC to estimate possible impact of RGC death on the structural integrity of these cells. Fig 2E shows maximum projections of the images of two DSACs from eyes without (E1) and with (E2) ONC while Fig 2F shows the tracing results of these two cells. The images show clearly that the DSAC from the eye after ONC has significantly reduced dendritic density.

**Fig 3:**
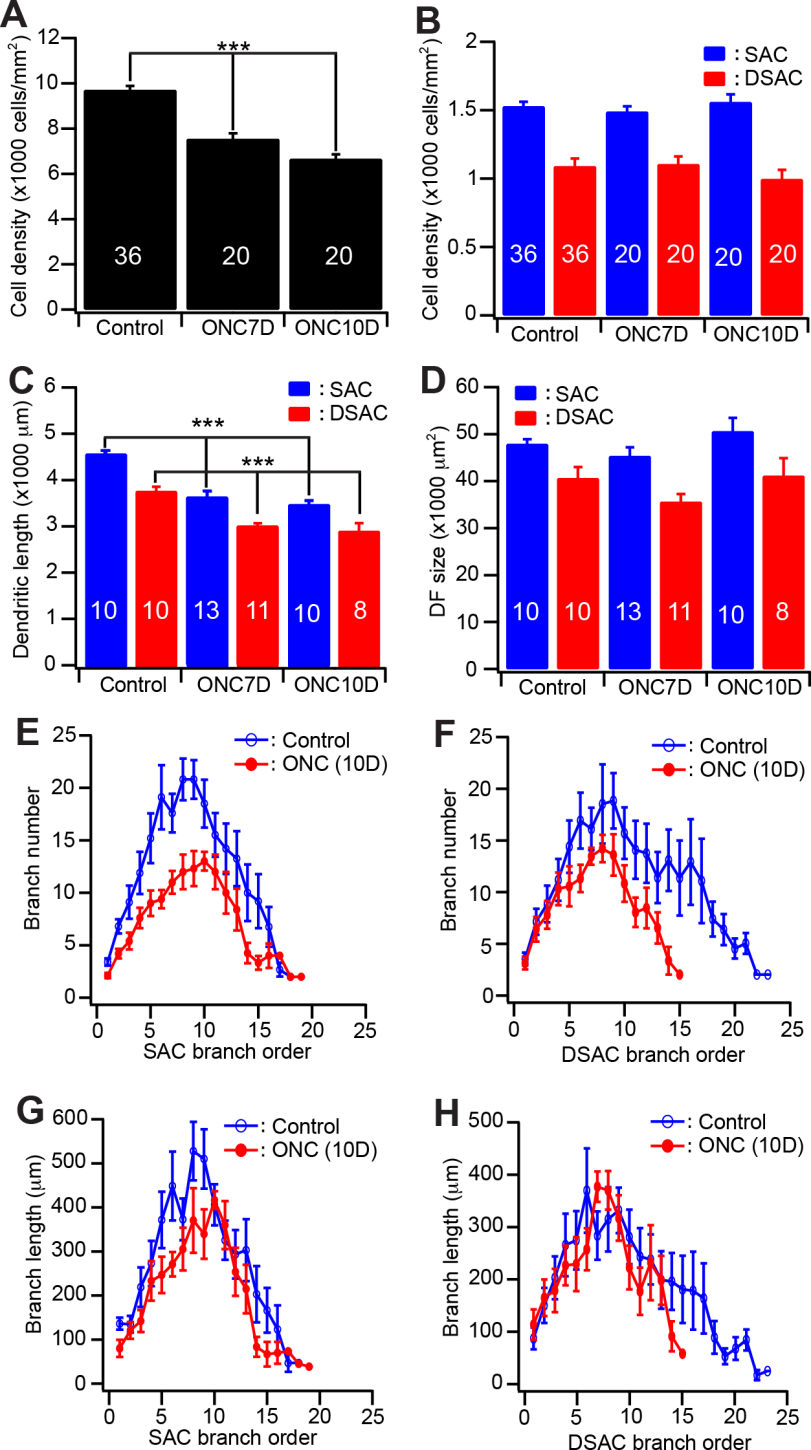
Quantitative analysis of the dendritic structure of SACs/DSACs after ONC. (**A**) The average densities of DAPI stained nuclei of GCL of mice with and without ONC. (**B**) The average densities of anti-ChAT antibody stained SACs and DSACs before and after ONC. (**C**) Average total length of dendrites of SACs and DSACs of mice before and after ONC. (**D**) Average size of dendritic field of SACs and DSACs of mice before and after ONC. (**E**) The number of dendritic branch as a function of dendritic order of SACs before and 10 days after ONC. (**F**) The number of dendritic branch as a function of dendritic order of DSACs before and 10 days after ONC. (**G**) The length of dendritic branch as a function of dendritic order of SACs before and 10 days after ONC. (**H**) The length of dendritic branch as a function of dendritic order of DSACs before and 10 days after ONC. The numbers in the columns of panels A-B indicate number of images analyzed. The numbers in the columns of panels C-D indicate the numbers of cells analyzed.

Quantitatively, the density of DAPI stained cells in GCL is reduced by 22% and 31% 7 and 10 days after ONC, respectively (Fig 3A), demonstrating that ONC caused significant death of neurons in the GCL. However, 10 days after ONC the densities of SACs and DSACs of the same retinas remained the similar levels observed in eyes without ONC. The average densities of DSACs and SACs 10 days after ONC are not significantly different from those found in the control eyes without ONC (Fig 3B). Therefore, SACs/DSACs do not die concurrently with the death of RGCs after ONC. On the other hand, the dendritic structures of SACs and DSACs are significantly altered shortly after ONC. Specifically, the dendritic lengths of both DSACs and SACs are decreased by 20% and 24% 10 days after ONC (Fig 3C) without a significant change in the sizes of their dendritic fields (Fig 3D. Also, see S2 Table for more details of statistic tests). These results demonstrated that RGC death after ONC is associated with quick dendritic reorganization of SACs/DSACs. Notably the impairment of the dendrites of SACs and DSACs are different after ONC. SACs preferentially decrease in number and length of proximal dendritic braches (Fig 3E–3G) while the DSACs have reduced number and length of distal dendritic branches (Fig 3F–3H).

### CD3ζ mutation impairs the dendrites of SACs/DSACs

**Fig 4:**
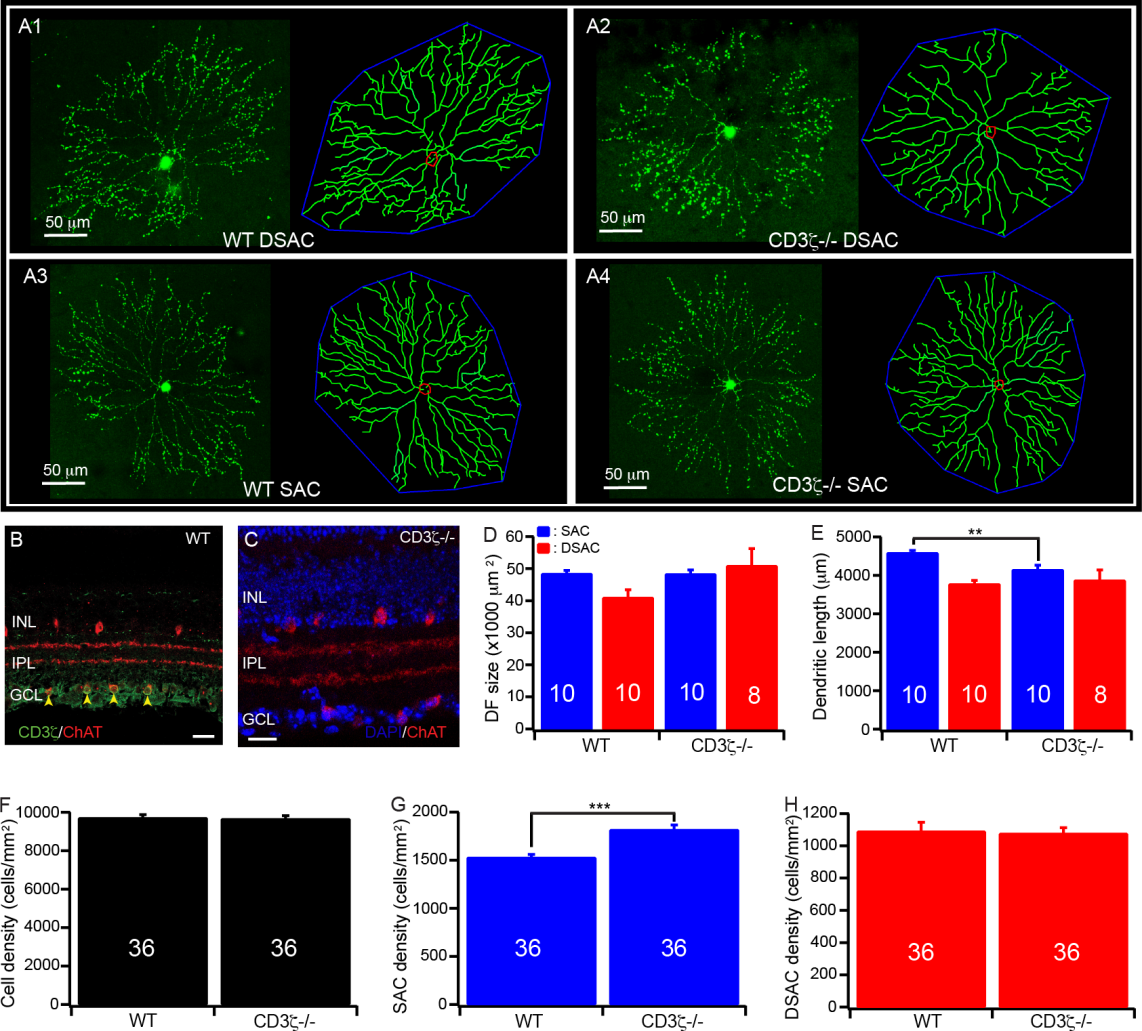
Mutation of CD3ζ impairs the development of SACs/DSACs. (**A**) Representative maximum projection images with the dendritic tracing of SACs and DSACs of WT and CD3ζ-/- mice. (**B**) A cross section of the retina of a WT mouse showing the co-labeling of ChAT (red) and CD3ζ (green) antibody staining (scale bar: 20μm). (**C**) A cross section of the retina a CD3ζ-/- mouse showing anti-ChAT antibody staining of SACs/DSACs (red) and DAPI (blue) staining of the retina (scale bar: 20μm). (**D**) Average size of the dendritic field of SACs and DSACs in both WT and CD3ζ-/- mice. (**E**) The total dendritic length of SACs and DSACs in both WT and CD3ζ-/- mice. (**F**) The average densities of DAPI stained nuclei of GCL in both WT and CD3ζ-/- mice. (**G**) The average densities of SACs in both WT and CD3ζ-/- mice. (**H**) The average densities of DSACs in both WT and CD3ζ-/- mice. The numbers in the columns of panels D-E indicate the number of cells analyzed. The numbers in the columns of panels F-H indicate the numbers of images analyzed.

We have previously shown that CD3ζ is expressed by RGCs and displaced amacrine cells in the mouse retina, including DSACs but not SACs (Fig 4B), and mutation of CD3ζ impairs dendritic development of RGCs [20]. Therefore, we investigated whether mutation of CD3ζ also impairs the dendrites of DSACs. Accordingly, we generated the CreER-ChAT:Stop-YFP:CD3ζ-/- triple transgenic mice, imaged and traced the dendrites of both SACs and DSACs in these mice. Fig 4A shows representative images of SACs and DSACs of CreER-ChAT:Stop-YFP and CreER-ChAT:Stop-YFP:CD3ζ-/- mice and the tracing results of these cells. It is evident that the typical ramification pattern of the dendritic plexus of SACs/DSACs in the IPL remains unaffected in the CreER-ChAT:Stop-YFP:CD3ζ-/- mice (Fig 4C). However, a detailed quantitative analysis of the dendrites of SACs/DSACs shows that the size of dendritic field of DSACs is increased by 38% in CreER-ChAT:Stop-YFP:CD3ζ-/- mice (Fig 4D) without significant change of the total dendritic length (Fig 4E). On the other hand, the size of the dendritic field of the SACs is not affected by CD3ζ mutation (Fig 4D) but the total dendritic length of these cells is decreased by 10% (Fig 4E). Furthermore, the density of SACs is increased by 19% in CD3ζ mutants (Fig 4G) while the densities of both the total cell count in the GCL, and the DSACs are not affected by CD3ζ mutation (Fig 4F–4H. Also, see S3 Table for more details of statistic tests.). These results demonstrated that CD3ζ mutation differentially impairs the dendrites of SACs and DSACs.

### CD3ζ mutation has no effect on cell death or dendritic reorganization of retinal neurons after ONC

**Fig 5:**
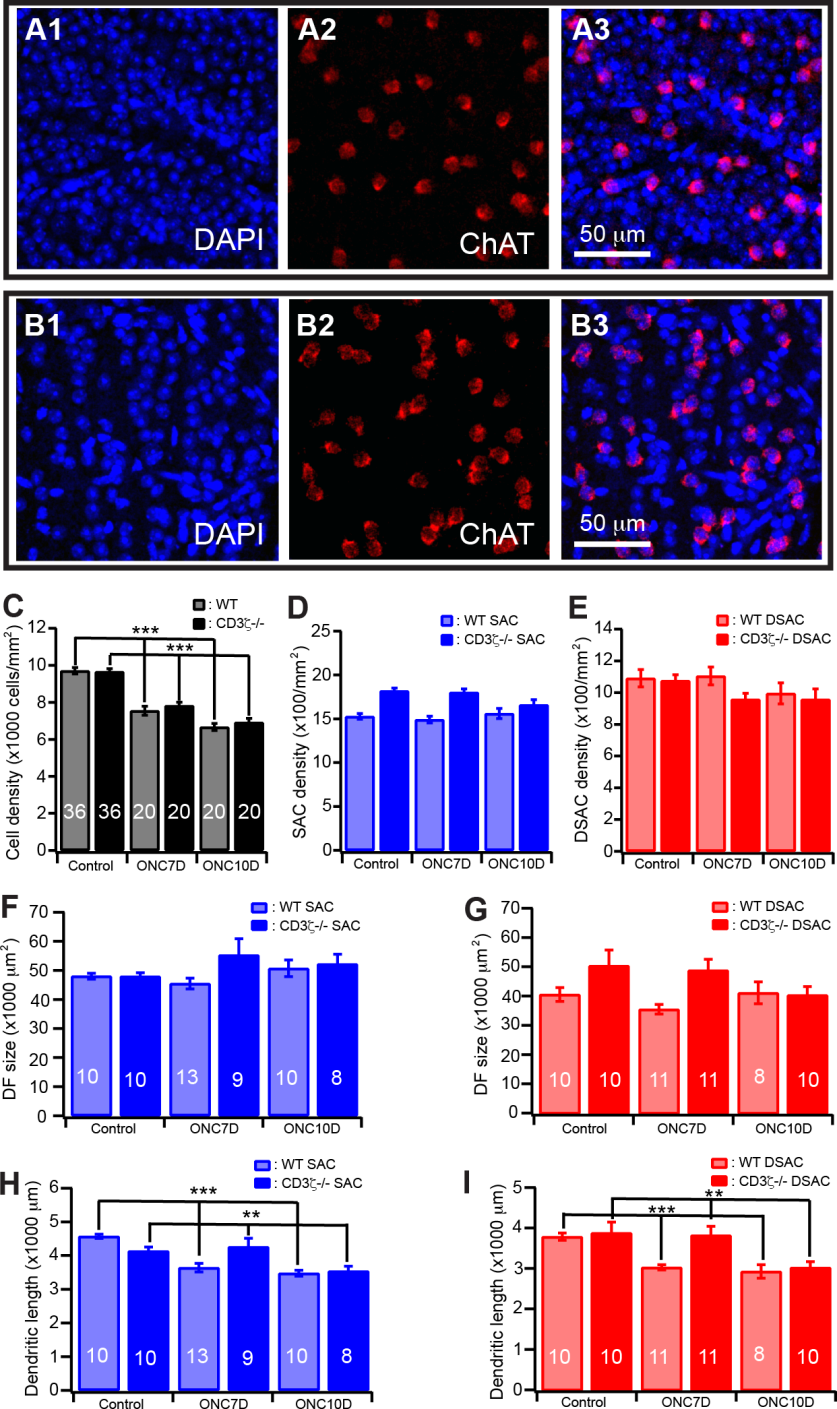
Mutation of CD3ζ does not alter the dendritic reorganization of SACs/DSACs after ONC. (**A**) Magnification from whole mount retina of a CD3ζ-/- mouse without ONC showing the density of DAPI stained nuclei in the GCL (A1), the density of anti-ChAT stained DSACs (A2) and the overlay of the DAPI and anti-ChAT stainings (A3). (**B**) Magnification from whole mount retina of a CD3ζ-/- mouse with ONC showing the density of DAPI stained nuclei in the GCL (B1), the density of anti-ChAT stained DSACs (B2) and the overlay of the DAPI and anti-ChAT stainings (B3). (**C**) The densities of DAPI stained nuclei of GCL of WT and CD3ζ-/- mice before and 10 days after ONC. (**D**) The densities of SACs of WT and CD3ζ-/- mice before and 10 days after ONC. (**E**) The densities of DSACs of WT and CD3ζ-/- mice before and 10 days after ONC. (**F**) The size of dendritic field of SACs of WT and CD3ζ-/- mice before and 10 days after ONC. (**G**) The size of dendritic field of DSACs of WT and CD3ζ-/- mice before and 10 days after ONC. (**H**) The total length of dendrites of SACs of WT and CD3ζ-/- mice before and 10 days after ONC. (**I**) The total length of dendrites of DSACs of WT and CD3ζ-/- mice before and 10 days after ONC. The numbers in the columns of panels B-D indicate number of images analyzed. The numbers in the columns of panels E-H indicate the numbers of cells analyzed.

Because MHCI-deficient mice have shown either more extensive neuronal injury [14–16] or protection of neurons from injury [18] and mutation of CD3ζ has impaired dendritic structure of RGCs of mice [11, 20], we thought to determine if CD3ζ regulates death of RGCs and dendritic reorganization of SACs/DSACs after ONC. Accordingly, the optic nerve of CreER-ChAT:Stop-YFP:CD3ζ-/- mice was crushed and the density of cells in the GCL and the dendrites of SACs and DSACs were qualified. Fig 5A and 5B shows anti-ChAT antibody and DAPI stained cells and nuclei in the GCL of CreER-ChAT:Stop-YFP:CD3ζ-/- mice without (5A) and with (5B) ONC. Similar to CreER-ChAT:Stop-YFP mice, the cell density in the GCL of CreER-ChAT:Stop-YFP:CD3ζ-/- mice is reduced by 28% 10 days after ONC versus 31% in CreER-ChAT:Stop-YFP mice (Fig 5C). On the other hand, the densities of SACs and DSACs in the same retina are not affected by ONC (Fig 5D and 5E). Furthermore, the dendritic length of SACs and DSACs of CreER-ChAT:Stop-YFP:CD3ζ-/- mice are reduced by 22% and 14%, respectively, after ONC (Fig 5H and 5I), while the sizes of the dendritic fields of both SACs and DSACs of CreER-ChAT:Stop-YFP:CD3ζ-/- mice are not altered after ONC (Fig 5F and 5G. Also see S4 and S5 Tables for more details of statistic tests.). Therefore, CD3ζ mutation has no effect on the cell death or dendritic reorganization of retinal neurons after ONC.

## Discussion

In this study, we have for the first time demonstrated three significant findings. First, RGC death after ONC is associated with immediate dendritic loss and reorganization of both SACs and DSACs, suggesting retrograde synaptic degeneration of these cells. Second, deletion of CD3ζ not only affects the dendritic development of DSACs, which express CD3ζ, it also affects the dendritic development of SAC and their density, which do not express CD3ζ. These results strongly support the possibility that CD3ζ is active and regulates the development of DSACs cell autonomously and SACs through synaptic circuits during postnatal development. Third, in contrast to previous findings that activation of MHCI or its receptors promote functional recovery of motor neurons and mutation of MHCI or its receptors prevent death of brain neurons, we show that mutation of CD3ζ have no effect on RGC death or dendritic reorganization of SACs/DSACs after ONC, suggesting that the immune molecules expressed by neurons might participate in neuronal pathogenesis or protection in a cell type specific manner.

### SACs/DSACs dendritic reorganization after ONC

The effects of RGC death on amacrine cells have been contradictory. Some previous studies showed that injury of RGC axons in neonatal rats or RGC elimination in ferret altered the number of GABAergic and glycinergic amacrine cells [1–3], while other studies showed that RGC elimination by optic nerve crush or transection had no effect on the number of SACs/DSACs of rats [4, 6] and no other cell type than RGCs in the GCL is as severely affected as to die during experimental glaucoma [38–40]. Our study showed that ONC caused significant decrease in the number of cells in the GCL without changing of the number of SACs/DSACs in mice. In addition, we show that SACs/DSACs rapidly lose dendrites after ONC. To the best of our knowledge, similar dendritic reorganization of SACs/DSACs after ONC has not been described previously.

Normally, SACs/DSACs have a strikingly symmetric dendritic morphology. The dendrites of DSACs sharply stratify within the ON sublamina of the IPL and the dendrites of SACs stratify within the OFF sublamina of the IPL [41–44]. In contrast to the symmetric dendritic morphology, the synaptic connections of SACs/DSACs are radially polarized with the synaptic inputs located across the entire dendritic arbor but their outputs restricted to the distal dendrites [45]. Physiologically, SACs/DSACs release both the excitatory neurotransmitter, acetylcholine, and the inhibitory neurotransmitter, GABA [46–49]. The GABAergic outputs of SACs/DSACs provide critical inhibition to the generation of direction selectivity of RGCs [50].

In our study, SACs preferentially lose their proximal dendrites while DSACs preferentially lose their distal dendrites after ONC. Because RGCs are the only cells directly injured by ONC, the dendritic reorganization of SACs and DSACs is most likely the result of a retrograde synaptic degeneration after RGC death. However, because the synaptic outputs of SACs/DSACs are restricted to the distal dendrites [45], the selective loss of proximal dendrites of SACs suggests an injury to their input cells.

It was postulated that the loss of RGCs might be directly responsible for the reduction of the number of amacrine cells and the effects appear to be limited to the amacrine cells that directly synapse with RGCs [2]. Mechanistically, RGCs might provide a source of trophic support for amacrine cells to stabilize their dendrites and synaptic connections with RGCs. Loss of RGC support will result in the removal of the synaptic connections between RGCs and amacrine cells. Consistent with this possibility, both RGCs and amacrine cells were found to synthesize brain-derived neurotrophic factor (BDNF) [51], which could regulate the survival and synaptic formation of retinal neurons [52–56]. With this consideration, the selective loss of distal dendrites of DSACs could be the result of a loss of RGC targets. If this is the case, RGCs might be required to maintain the normal dendritic architecture of DSACs although the dendritic development of SACs/DSACs is independent of RGCs [57].

### T-cell receptor component, CD3ζ, in SACs/DSACs development

Previous studies have shown that MHCI is expressed by neurons in both GCL and INL of mouse retina, while CD3ζ is expressed by RGCs and DSACs [11, 20]. The phenotypic defects of CD3ζ mutation on RGC axon and function highly resemble that of MHCI mutants [11, 20]. Because TCR functions as the major receptor of MHCI in the immune system and CD3ζ is the key component of TCR complex [20], it is possible that MHCI might regulate RGC structure and function through TCR in retina. In the T-cells, engagement of MHCI with TCR activates several downstream molecular cascades, leading to activation of RAS, mobilization of intracellular calcium, activation of PKC, and reorganization of the actin-based cytoskeleton [19]. These molecular cascades are also expressed in the CNS and implicated in the activity-dependent synaptic plasticity of neurons [58]. However, the ligand-receptor relationship between MHCI and TCR has not been well established in CNS and PirB, the putative neuronal MHCI receptor in brain, has not been detected in retina.

Several findings of our study are noteworthy. First, the mutation of CD3ζ impaired the dendrites of both SACs and DSACs but the effects on DSACs and SACs are different. Because DSACs express CD3ζ but SACs do not, the effects of CD3ζ mutation on SACs have to be indirect. However, it is possible that the effects of CD3ζ mutation on DSACs might be direct, indirect or a combination of both direct and indirect effects. It is possible that the dendritic development of SACs dependents upon glutamatergic synaptic activity. Consistently, both the spontaneous glutamatergic synaptic activity and light evoked responses of the retina of CD3ζ mutants are significantly reduced during early postnatal development, which is associated with a reduced dynamic of RGC dendritic growth and elimination [20]. Second, the effects of CD3ζ mutation on the dendritic defects of DSACs and RGCs are different although both RGCs and DSACs express CD3ζ. RGCs of CD3ζ mutants have an increased number of dendritic branches and the total length of dendrites but normal dendritic field area [20], while the DSACs of CD3ζ mutants have enlarged dendritic field area but normal total length of dendrites and the number of dendritic branches. These differences suggest a possibility of cell type specific responses to CD3ζ mutation in the retina or maybe a combined cell autonomous effects and indirect effects on DSACs. Third, the density of SACs, but not DSACs, is increased in the CD3ζ mutants. Because the differentiation of SACs/DSACs occurs before birth and the retina of CD3ζ mutants showed no detectable structural and function defects until 10 day after birth [11, 20], it suggests that CD3ζ might participate in the program cells death of SACs, which occurs mostly before postnatal day 14 [59–60]. Overall, these results provided for the first time the roles of CD3ζ on the development of retinal amacrine cells.

### Potential effects of CD3ζ in neuronal degeneration under disease conditions

The effects of MHCI and its receptors on neuronal death and protection are contradictory. Several reports have shown that the motor neurons of mice with reduced MHCI expression have more extensive loss of synapses and reduced axon regeneration after nerve transection and increased susceptible to astrocyte-induced cell death. On the other hand, up-regulation of MHCI expression in motor neurons increased survival and promoted the recovery of motor performance [14–17]. Therefore, the activation of MHCI and its receptors appears to be beneficial for motor neuron survival and functional recovery. In contrast, another study showed that mice with MHCI and PirB knockout have smaller infarcts and enhanced motor recovery after stroke, less cell death after ischemia of hippocampus, and reduced astrocytic response after cerebral artery occlusion [18]. Thus, the activation of MHCI and its receptors appears to exacerbate brain injury after ischemia. In our study, mutation of CD3ζ has no effect on either the death of neurons in the GCL of retina or the dendritic reorganization of SACs/DSACs after ONC although mutation of CD3ζ impaired the proper development of the dendrites of both SACs and DSACs.

Several factors could contribute to this discrepancy. First, MHCI and its receptors could regulate neuron survival through more than one mechanism. For instance, MHCI and its receptors likely regulate motor neuron survival after spinal cord transection through a signal pathway within motor neurons [14, 17, 61], while the death of motor neurons in amyotrophic lateral sclerosis is thought to be the results of astrocytes induced toxicity [17]. Second, the effects of MHCI and its receptors could be cell type specific. This is consistent with the reports that activation of MHCI and its receptors on motor neurons stabilize synaptic connections, increase synapse formation, and limit secondary neuronal degeneration after spinal cord transection [14, 17, 61], while activation of MHCI and PirB limit axonal outgrowth in regeneration of CNS neurons after injury [62–65]. Third, different types of pathological insults might trigger different effects mediated by MHCI and its receptors. In PirB mutants, ischemia triggers an increase in the number of midline crossing fibers from the undamaged corticospinal tract into the denervated red nucleus [18], while spinal cord injury or traumatic brain injury does not affect axonal regeneration, functional recovery or axonal plasticity of neurons [66–67]. Finally, although both PirB and TCR could function as the receptors of neuronal MHCI [68], PirB is unlikely to be the MHCI receptor in retina because it has not been detected in the retina. Therefore, TCR or CD3ζ will most likely to be the receptor of MHCI in retina. However, whether MHCI could regulate the neuroprotection through CD3ζ has not been reported previously. Our results demonstrate that mutation of CD3ζ has no effect on RGC death and SACs/DSACs dendritic reorganization after ONC.

## Supporting information

S1 Table Dendritic structure and cell density of SACs and DSACs. The differences of dendritic structure and cell density between SACs and DSACs of untreated wild type mice were statistically tested using t-tests. The mean, standard error (SE), number of cells (n) for dendritic structure and number of views (n, four views per retina) for cell density calculation of each group as well as the t and p values of the t-tests are shown here.
(DOCX)

S2 Table Retinal cell density and dendritic structure of SACs and DSACs with or without ONC. The differences in the dendritic structure of SACs and DSACs and the cell densities of SACs, DSACs and cells in GCL of wild type mice under three conditions (before ONC, 7 days after ONC and 10 days after ONC) were statistically examined using student t-tests. The mean, standard error (SE), number of cells (n) for dendritic structure and the number of views (n, four views per retina) for cell density calculation of each group as well as the t and p values are shown here. The t and p values listed in the same row of “7D after ONC” and “10D after ONC” are the results of comparison with control (Before ONC). The row “7D versus 10D” lists the t and p values of comparison between “7D after ONC” and “10D after ONC”.
(DOCX)

S3 Table Dendritic structure and cell density of starburst amacrine cells of CD3ζ mutants. The differences in the dendritic structure of SACs and DSACs and the cell densities of SACs, DSACs and cells in GCL between wild type and CD3ζ-/- mice were statistically tested using t-tests. The mean, standard error (SE), number of cells (n) for dendritic structure and number of views (n, four views per retina) for cell density calculation of each group as well as the t and p values of the t-tests are shown here.
(DOCX)

S4 Table Cell density and dendritic structure of SACs and DSACs of CD3ζ-/- mice after ONC. The differences in the dendritic structure of SACs and DSACs and the cell densities of SACs, DSACs and cells in GCL of CD3ζ-/- mice under three conditions (before ONC, 7 days after ONC and 10 days after ONC) were statistically examined using student t-tests. The mean, standard error (SE), number of cells (n) for dendritic structure and number of views (n, four views per retina) for cell density calculation of each group as well as the t and p values of the t-tests are shown here.
(DOCX)

S5 Table Comparison of the dendritic structure and cell density of starburst amacrine cells of WT and CD3ζ mutants after ONC. The differences in the dendritic structure of SACs and DSACs and the cell densities of SACs, DSACs and cells in GCL of CD3ζ-/- mice under two conditions (7 days after ONC and 10 days after ONC) were compared with mice without CD3ζ mutation after ONC. The mean, standard error (SE), number of cells (n) for dendritic structure and number of views (n, four views per retina) for cell density calculation of each group as well as the t and p values of the t-tests are shown here.
(DOCX)
