# Correction: The effects of immune protein CD3ζ development and degeneration of retinal neurons after optic nerve injury

**DOI:** 10.1371/journal.pone.0198770

**Published:** 2018-06-01

**Authors:** Tao He, Xavier Mortensen, Ping Wang, Ning Tian

There is an error in affiliation 1 for the first author, Tao He. Affiliation 1 should be: Eye Center Renmin Hospital of Wuhan University Wuhan, Hubei, PR China.
