# Supplementary material for: The effects of immune protein CD3ζ development and degeneration of retinal neurons after optic nerve injury
Source: PLoS One. 2017 Apr 25;12(4):e0175522. doi: 10.1371/journal.pone.0175522 (PMC5404868; doi:10.1371/journal.pone.0175522)
Supplement: S1 Table — The differences of dendritic structure and cell density between SACs and DSACs of untreated wild type mice were statistically tested using t-tests. The mean, standard error (SE), number of cells (n) for dendritic structure and number of views (n, four views per retina) for cell density calculation of each group as well as the t and p values of the t-tests are shown here. (DOCX) [file pone.0175522.s001.docx]

**S1 Table 1. Dendritic structure and cell density of SACs and DSACs**

| Cell types | Mean | SE | n | t | p |
| --- | --- | --- | --- | --- | --- |
| Dendritic field size (μm^2^) | | | | | |
| SAC | 47930 | 997 | 10 | -2.863 | 0.0103 |
| DSAC | 40608 | 2355 | 10 |  |  |
| Dendritic length (μm) | | | | | |
| SAC | 4574 | 61 | 10 | -7.365 | <0.0001 |
| DSAC | 3770 | 90 | 10 |  |  |
| Cell density (cells/mm^2^) | | | | | |
| SAC | 1529 | 33 | 36 | -6.792 | <0.0001 |
| DSAC | 1091 | 55 | 36 |  |  |
| Number of dendritic branch | | | | | |
| SAC | 209 | 5.9 | 10 | 0.61 | 0.55 |
| DSAC | 200 | 12.6 | 10 |  |  |
| Average dendritic branch length (μm) | | | | | |
| SAC | 312 | 14 | 10 | -2.58 | 0.0189 |
| DSAC | 235 | 26.4 | 10 |  |  |

The differences of dendritic structure and cell density between SACs and DSACs of untreated wild type mice were statistically tested using t-tests. The mean, standard error (SE), number of cells (n) for dendritic structure and number of views (n, four views per retina) for cell density calculation of each group as well as the t and p values of the t-tests are shown here.
