# Supplementary material for: The effects of immune protein CD3ζ development and degeneration of retinal neurons after optic nerve injury
Source: PLoS One. 2017 Apr 25;12(4):e0175522. doi: 10.1371/journal.pone.0175522 (PMC5404868; doi:10.1371/journal.pone.0175522)
Supplement: S2 Table — The differences in the dendritic structure of SACs and DSACs and the cell densities of SACs, DSACs and cells in GCL of wild type mice under three conditions (before ONC, 7 days after ONC and 10 days after ONC) were statistically examined using student t-tests. The mean, standard error (SE), number of cells (n) for dendritic structure and the number of views (n, four views per retina) for cell density calculation of each group as well as the t and p values are shown here. The t and p values listed in the same row of “7D after ONC” and “10D after ONC” are the results of comparison with control (Before ONC). The row “7D versus 10D” lists the t and p values of comparison between “7D after ONC” and “10D after ONC”. (DOCX) [file pone.0175522.s002.docx]

**S2 Table 2. Retinal cell density and dendritic structure of SACs and DSACs with or without ONC**

|  | Mean | SE | n | t | p |
| --- | --- | --- | --- | --- | --- |
| GCL cell density (cells/mm^2^) | | | | | |
| Before ONC | 9707 | 171 | 36 |  |  |
| 7D after ONC | 7548 | 243 | 20 | 7.394 | <0.0001 |
| 10D after ONC | 6668 | 192 | 20 | 11.244 | <0.0001 |
| 7D versus 10D |  |  |  | -2.84 | 0.0072 |
| Density of SACs (cells/mm^2^) | | | | | |
| Before ONC | 1529 | 33 | 36 |  |  |
| 7D after ONC | 1490 | 38 | 20 | 0.729 | 0.4694 |
| 10D after ONC | 1561 | 57 | 20 | -0.522 | 0.6041 |
| 7D versus 10D |  |  |  | 1.031 | 0.3089 |
| Density of DSACs (cells/mm^2^) | | | | | |
| Before ONC | 1091 | 55 | 36 |  |  |
| 7D after ONC | 1105 | 57 | 20 | -0.165 | 0.8694 |
| 10D after ONC | 995 | 67 | 20 | 1.068 | 0.2904 |
| 7D versus 10D |  |  |  | -1.251 | 0.2185 |
| Dendritic length of DSACs (μm) | | | | | |
| Before ONC | 3770 | 90 | 10 |  |  |
| 7D after ONC | 3018 | 65 | 11 | 6.858 | <0.0001 |
| 10D after ONC | 2920 | 164 | 8 | 4.794 | 0.0002 |
| 7D versus 10D |  |  |  | -0.616 | 0.546 |
| Dendritic length of SACs (μm) | | | | | |
| Before ONC | 4574 | 61 | 10 |  |  |
| 7D after ONC | 3642 | 126 | 13 | 6.031 | <0.0001 |
| 10D after ONC | 3477 | 87 | 10 | 10.301 | <0.0001 |
| 7D versus 10D |  |  |  | -1.011 | 0.3236 |
| Dendritic field size of DSACs (μm^2^) | | | | | |
| Before ONC | 40608 | 2355 | 10 |  |  |
| 7D after ONC | 35571 | 1630 | 11 | 1.786 | 0.09 |
| 10D after ONC | 41152 | 3750 | 8 | -0.128 | 0.8998 |
| 7D versus 10D |  |  |  | 1.507 | 0.1501 |
| Dendritic field size of SACs (μm^2^) | | | | | |
| Before ONC | 47391 | 997 | 10 |  |  |
| 7D after ONC | 45358 | 1829 | 13 | 1.133 | 0.2699 |
| 10D after ONC | 50613 | 2841 | 10 | -0.891 | 0.3849 |
| 7D versus 10D |  |  |  | 1.62 | 0.1202 |

The differences in the dendritic structure of SACs and DSACs and the cell densities of SACs, DSACs and cells in GCL of wild type mice under three conditions (before ONC, 7 days after ONC and 10 days after ONC) were statistically examined using student t-tests. The mean, standard error (SE), number of cells (n) for dendritic structure and the number of views (n, four views per retina) for cell density calculation of each group as well as the t and p values are shown here. The t and p values listed in the same row of “7D after ONC” and “10D after ONC” are the results of comparison with control (Before ONC). The row “7D versus 10D” lists the t and p values of comparison between “7D after ONC” and “10D after ONC”.
