# Supplementary material for: The effects of immune protein CD3ζ development and degeneration of retinal neurons after optic nerve injury
Source: PLoS One. 2017 Apr 25;12(4):e0175522. doi: 10.1371/journal.pone.0175522 (PMC5404868; doi:10.1371/journal.pone.0175522)
Supplement: S3 Table — The differences in the dendritic structure of SACs and DSACs and the cell densities of SACs, DSACs and cells in GCL between wild type and CD3ζ-/- mice were statistically tested using t-tests. The mean, standard error (SE), number of cells (n) for dendritic structure and number of views (n, four views per retina) for cell density calculation of each group as well as the t and p values of the t-tests are shown here. (DOCX) [file pone.0175522.s003.docx]

**S3 Table 3. Dendritic structure and cell density of starburst amacrine cells of CD3ζ mutants**

| Cell type | Mean | SE | n | t | p |
| --- | --- | --- | --- | --- | --- |
| Dendritic field size of DSACs (μm^2^) | | | | | |
| WT | 40608 | 2355 | 10 | 1.813 | 0.0887 |
| CD3**ζ**-/- | 50376 | 5288 | 8 |  |  |
| Dendritic length of DSACs (μm) | | | | | |
| WT | 3770 | 90 | 10 | 0.37 | 0.716 |
| CD3**ζ**-/- | 3865 | 264 | 8 |  |  |
| Dendritic field size of SACs (μm^2^) | | | | | |
| WT | 47931 | 997 | 10 | -0.058 | 0.9541 |
| CD3**ζ**-/- | 47839 | 1213 | 10 |  |  |
| Dendritic length of SACs (μm) | | | | | |
| WT | 4574 | 61 | 10 | -3.301 | 0.004 |
| CD3**ζ**-/- | 4137 | 117 | 10 |  |  |
| GCL cell density (cells/mm^2^) | | | | | |
| WT | 9707 | 171 | 36 | -0.198 | 0.844 |
| CD3**ζ**-/- | 9661 | 164 | 36 |  |  |
| Density of SACs (cells/mm^2^) | | | | | |
| WT | 1529 | 33 | 36 | 4.918 | <0.0001 |
| CD3**ζ**-/- | 1818 | 49 | 36 |  |  |
| Density of DSACs (cells/mm^2^) | | | | | |
| WT | 1091 | 55 | 36 | -0.215 | 0.8307 |
| CD3**ζ**-/- | 1077 | 36 | 36 |  |  |

The differences in the dendritic structure of SACs and DSACs and the cell densities of SACs, DSACs and cells in GCL between wild type and CD3ζ-/- mice were statistically tested using t-tests. The mean, standard error (SE), number of cells (n) for dendritic structure and number of views (n, four views per retina) for cell density calculation of each group as well as the t and p values of the t-tests are shown here.
