# Supplementary material for: The effects of immune protein CD3ζ development and degeneration of retinal neurons after optic nerve injury
Source: PLoS One. 2017 Apr 25;12(4):e0175522. doi: 10.1371/journal.pone.0175522 (PMC5404868; doi:10.1371/journal.pone.0175522)
Supplement: S4 Table — The differences in the dendritic structure of SACs and DSACs and the cell densities of SACs, DSACs and cells in GCL of CD3ζ-/- mice under three conditions (before ONC, 7 days after ONC and 10 days after ONC) were statistically examined using student t-tests. The mean, standard error (SE), number of cells (n) for dendritic structure and number of views (n, four views per retina) for cell density calculation of each group as well as the t and p values of the t-tests are shown here. (DOCX) [file pone.0175522.s004.docx]

**S4 Table 4. Cell density and dendritic structure of SACs and DSACs of CD3ζ-/- mice after ONC**

| Cell type | Mean | SE | n | t | p |
| --- | --- | --- | --- | --- | --- |
| GCL cell density of CD3**ζ**-/- mice (cells/mm^2^) | | | | | |
| Before ONC | 9661 | 164 | 36 |  |  |
| 7D after ONC | 7825 | 187 | 20 | 6.654 | <0.0001 |
| 10D after ONC | 6920 | 209 | 20 | 10.171 | <0.0001 |
| 7D versus 10D |  |  |  | -3.141 | 0.0035 |
| Density of SACs of CD3**ζ**-/- mice (cells/mm^2^) | | | | | |
| Before ONC | 1818 | 49 | 36 |  |  |
| 7D after ONC | 1803 | 64 | 20 | 0.18 | 0.8577 |
| 10D after ONC | 1660 | 87 | 20 | 1.716 | 0.0919 |
| 7D versus 10D |  |  |  | -1.321 | 0.1944 |
| Density of DSACs of CD3**ζ**-/- mice (cells/mm^2^) | | | | | |
| Before ONC | 1077 | 36 | 36 |  |  |
| 7D after ONC | 958 | 38 | 20 | 2.129 | 0.0378 |
| 10D after ONC | 958 | 65 | 20 | 1.745 | 0.0867 |
| 7D versus 10D |  |  |  | -1.26E-9 | >0.9999 |
| Dendritic field size of DSACs of CD3**ζ**-/- mice (μm^2^) | | | | | |
| Before ONC | 50376 | 5288 | 8 |  |  |
| 7D after ONC | 48898 | 3669 | 11 | 0.238 | 0.815 |
| 10D after ONC | 40445 | 2845 | 10 | 1.748 | 0.0996 |
| 7D versus 10D |  |  |  | -1.794 | 0.0887 |
| Dendritic length of DSACs of CD3**ζ**-/- mice (μm) | | | | | |
| Before ONC | 3865 | 264 | 8 |  |  |
| 7D after ONC | 3807 | 254 | 11 | 0.17 | 0.8668 |
| 10D after ONC | 3017 | 142 | 10 | 2.994 | 0.0086 |
| 7D versus 10D |  |  |  | -3.012 | 0.0072 |
| Dendritic field size of SACs of CD3**ζ**-/- mice (μm^2^) | | | | | |
| Before ONC | 47839 | 1213 | 10 |  |  |
| 7D after ONC | 55147 | 5555 | 9 | -1.351 | 0.1942 |
| 10D after ONC | 52119 | 3326 | 8 | -1.316 | 0.2066 |
| 7D versus 10D |  |  |  | -0.453 | 0.6573 |
| Dendritic length of SACs of CD3**ζ**-/- mice (μm) | | | | | |
| Before ONC | 4137 | 117 | 10 |  |  |
| 7D after ONC | 4261 | 250 | 9 | -0.466 | 0.6474 |
| 10D after ONC | 3539 | 144 | 8 | 3.254 | 0.005 |
| 7D versus 10D |  |  |  | -2.42 | 0.0287 |

The differences in the dendritic structure of SACs and DSACs and the cell densities of SACs, DSACs and cells in GCL of CD3ζ-/- mice under three conditions (before ONC, 7 days after ONC and 10 days after ONC) were statistically examined using student t-tests. The mean, standard error (SE), number of cells (n) for dendritic structure and number of views (n, four views per retina) for cell density calculation of each group as well as the t and p values of the t-tests are shown here.
