# Supplementary material for: The effects of immune protein CD3ζ development and degeneration of retinal neurons after optic nerve injury
Source: PLoS One. 2017 Apr 25;12(4):e0175522. doi: 10.1371/journal.pone.0175522 (PMC5404868; doi:10.1371/journal.pone.0175522)
Supplement: S5 Table — The differences in the dendritic structure of SACs and DSACs and the cell densities of SACs, DSACs and cells in GCL of CD3ζ-/- mice under two conditions (7 days after ONC and 10 days after ONC) were compared with mice without CD3ζ mutation after ONC. The mean, standard error (SE), number of cells (n) for dendritic structure and number of views (n, four views per retina) for cell density calculation of each group as well as the t and p values of the t-tests are shown here. (DOCX) [file pone.0175522.s005.docx]

**S5 Table 5. Comparison of the dendritic structure and cell density of starburst amacrine cells of WT and CD3ζ mutants after ONC**

| Cell type | Mean | SE | n | t | p |
| --- | --- | --- | --- | --- | --- |
| Dendritic field size of DSACs 7D after ONC (μm^2^) | | | | | |
| WT | 35571 | 1630 | 11 | 3.32 | 0.0034 |
| CD3**ζ**-/- | 48898 | 3669 | 11 |  |  |
| Dendritic length of DSACs 7D after ONC (μm) | | | | | |
| WT | 3018 | 65 | 11 | 3.529 | 0.0021 |
| CD3**ζ**-/- | 3807 | 254 | 11 |  |  |
| Dendritic field size of SACs 7D after ONC (μm^2^) | | | | | |
| WT | 45358 | 1829 | 13 | 1.927 | 0.0683 |
| CD3**ζ**-/- | 55147 | 5555 | 9 |  |  |
| Dendritic length of SACs 7D after ONC (μm) | | | | | |
| WT | 3642 | 126 | 13 | 2.413 | 0.0255 |
| CD3**ζ**-/- | 4261 | 250 | 9 |  |  |
| GCL cell density 7D after ONC (cells/mm^2^) | | | | | |
| WT | 7548 | 243 | 20 | 0.864 | 0.3973 |
| CD3**ζ**-/- | 7825 | 187 | 20 |  |  |
| Density of SACs 7D after ONC (cells/mm^2^) | | | | | |
| WT | 1490 | 38 | 20 | 4.19 | 0.0002 |
| CD3**ζ**-/- | 1803 | 64 | 20 |  |  |
| Density of DSACs 7D after ONC (cells/mm^2^) | | | | | |
| WT | 1105 | 57 | 20 | -2.153 | 0.0377 |
| CD3**ζ**-/- | 958 | 38 | 20 |  |  |
| Dendritic field size of DSACs 10D after ONC (μm^2^) | | | | | |
| WT | 41152 | 3750 | 8 | -0.153 | 0.8802 |
| CD3**ζ**-/- | 40445 | 2845 | 10 |  |  |
| Dendritic length of DSACs 10D after ONC (μm) | | | | | |
| WT | 2920 | 164 | 8 | 0.45 | 0.6587 |
| CD3**ζ**-/- | 3017 | 142 | 10 |  |  |
| Dendritic field size of SACs 10D after ONC (μm^2^) | | | | | |
| WT | 50613 | 2841 | 10 | 0.346 | 0.7336 |
| CD3**ζ**-/- | 52119 | 3326 | 8 |  |  |
| Dendritic length of SACs 10D after ONC (μm) | | | | | |
| WT | 3477 | 87 | 10 | 0.383 | 0.7068 |
| CD3**ζ**-/- | 3539 | 144 | 8 |  |  |
| GCL cell density 10D after ONC (cells/mm^2^) | | | | | |
| WT | 6668 | 192 | 20 | 0.888 | 0.3799 |
| CD3**ζ**-/- | 6920 | 209 | 20 |  |  |
| Density of SACs 10D after ONC (cells/mm^2^) | | | | | |
| WT | 1561 | 57 | 20 | 0.962 | 0.3424 |
| CD3**ζ**-/- | 1660 | 87 | 20 |  |  |
| Density of DSACs 10D after ONC (cells/mm^2^) | | | | | |
| WT | 995 | 67 | 20 | -0.4 | 0.6916 |
| CD3**ζ**-/- | 958 | 65 | 20 |  |  |

The differences in the dendritic structure of SACs and DSACs and the cell densities of SACs, DSACs and cells in GCL of CD3ζ-/- mice under two conditions (7 days after ONC and 10 days after ONC) were compared with mice without CD3ζ mutation after ONC. The mean, standard error (SE), number of cells (n) for dendritic structure and number of views (n, four views per retina) for cell density calculation of each group as well as the t and p values of the t-tests are shown here.
